# Enhancing Transportation in Rural Communities Through Innovative Solutions

**DOI:** 10.1093/geroni/igaf122.1289

**Published:** 2025-12-31

**Authors:** Renee St. Louis, Renee St. Louis

## Abstract

Transportation remains one of the biggest barriers to maintaining health and quality of life in rural areas. While the challenges are familiar and have been well-documented, solutions are scarce. The presentations in this session collectively highlight successful examples of technology deployment, collaboration, and other innovative approaches to address challenges and improve accessibility for older adults. Marcus will discuss insights from an analysis of national data on older adults’ requests for rides in rural areas. Freund will describe the results of a study of ITNCountry’s national network of nonprofit community transportation services and the effects of the network on rider experience and trip volume. Jansen will present the results of an evaluation of Montachusett Regional Transit Authority (MART) initiatives to improve coordination among local Council on Aging transportation providers. Ramsey will summarize preliminary results of a pilot project in rural Iowa that has enabled medical patients to call public transportation rides on demand after their doctors’ visits have concluded. Brooks will introduce UZURV’s model for providing accessible, safety-conscious ridesharing services in rural areas and review insights from their rider data on remaining mobility challenges and opportunities. St. Louis will conclude as discussant, relating key themes across presentations and proposing next steps for research and practice. Transportation and Aging Interest Group Sponsored Symposium

